# *Moraxella catarrhalis* Adhesin UspA1-derived Recombinant Fragment rD-7 Induces Monocyte Differentiation to CD14+CD206+ Phenotype

**DOI:** 10.1371/journal.pone.0090999

**Published:** 2014-03-05

**Authors:** Qi Xie, Louise S. Brackenbury, Darryl J. Hill, Neil A. Williams, Xun Qu, Mumtaz Virji

**Affiliations:** 1 Institute of Basic Medical Sciences, Qilu Hospital, Shandong University, Jinan, Shandong, P.R. China; 2 School of Cellular and Molecular Medicine, University of Bristol, Bristol, United Kingdom; University of Toledo School of Medicine, United States of America

## Abstract

Circulating monocytes in the bloodstream typically migrate to other tissues and differentiate into tissue resident macrophages, the process being determined by the constituents of the microenvironments encountered. These may include microbes and their products. In this study, we investigated whether *Moraxella catarrhalis* Ubiquitous Surface Protein A1 (UspA1), known to bind to a widely expressed human cell surface receptor CEACAM1, influences monocyte differentiation as receptor engagement has been shown to have profound effects on monocytes. We used the recombinant molecules corresponding to the regions of UspA1 which either bind (rD-7; UspA1_527–665_) or do not bind (r6–8; UspA1_659–863_) to CEACAM1 and investigated their effects on CD206, CD80 and CD86 expression on freshly isolated human CD14+ monocytes from peripheral blood mononuclear cells (PBMC). Exposure to rD-7, but not r6–8, biased monocyte differentiation towards a CD14+CD206+ phenotype, with reduced CD80 expression. Monocytes treated with rD-7 also secreted high levels of IL-1ra and chemokine IL-8 but not IL-10 or IL-12p70. The effects of rD-7 were independent of any residual endotoxin. Unexpectedly, these effects of rD-7 were also independent of its ability to bind to CEACAM1, as monocyte pre-treatment with the anti-CEACAM antibody A0115 known to inhibit rD-7 binding to the receptor, did not affect rD-7-driven differentiation. Further, another control protein rD-7/D (a mutant form of rD-7, known not to bind to CEACAMs), also behaved as the parent molecule. Our data suggest that specific regions of *M. catarrhalis* adhesin UspA1 may modulate inflammation during infection through a yet unknown receptor on monocytes.

## Introduction

Monocytes and macrophages are both indispensable effector cells critical in regulation of inflammation and in non-specific innate immune responses, the first line of defence against invading bacteria [1]. They also regulate adaptive immunity in a cell-cell contact dependent manner or with secreted pro-inflammatory or anti-inflammatory cytokines and chemokines [2].

Monocytes are the circulating precursors of tissue macrophages and dendritic cells [3], [4], and macrophage colony-stimulating factor (M-CSF) is a potent monocyte/macrophage differentiation factor [5]. During such differentiation, signals initiated by different cytokines and specific surface receptors modify the process generating either classically activated M1 macrophages or alternatively activated M2 macrophages, which exhibit significant differences in receptor, cytokine and chemokine expression, and effector function. M1 macrophages are responsive to type 1 inflammatory cytokines as well as microbial products, whilst M2 macrophages can be induced by IL-4, IL-13, IL-1, IL-10 or hormones [6]. Certain studies have suggested that pathogens, for example *Yersinia sp.*, may have adapted mechanisms to alter the differentiation of monocytes via the expression of different virulence factors [7], [8]. M2 macrophages are characterized by their expression of specific membrane and chemokine receptors, such as mannose receptor (CD206), CD163, CXCR1, CXCR2, CCR2 and also by their secretion of several cytokines and chemokines, including IL-10, IL-1 receptor antagonist (IL-1ra), CCL17, CCL18, and CCL22 [9], [10]. CD206 is a transmembrane C-type lectin primarily expressed on the surface of macrophages and dendritic cells. It plays an important role in the innate immune response, recognising pathogens through mannose residues on their surfaces and mediating endocytosis and phagocytosis [11]. CD80 (B7-1) and CD86 (B7-2) belong to the B7 family of molecules and are expressed on antigen-presenting cells which provide co-stimulatory signals for T cell activation and survival [12]. Monocyte differentiation can also be regulated by immunoglobulin superfamily receptors [13] including members of the carcinoembryonic antigen (CEA) family.

Among the members of the CEA family, the CEA-related cell adhesion molecule 1 (CEACAM1), also known as CD66a, is expressed on several types of cell including human granulocytes and lymphocytes, endothelia, epithelia, extravillous trophoblasts and some tumour cells. It serves as a sophisticated signalling molecule which participates in regulating a variety of cellular activities [14], [15], [16], [17], [18], [19], [20], [21], [22], [23]. A vital role for CEACAM1, the only member of the CEA family found on monocytes [24], [25], in the survival and function of monocytes has been suggested; and CEACAM1-dependent regulation of primary CD14+ monocyte survival and control of angiogenesis in a murine inflammation model has also been reported [16], [24]. In addition, on a number of human cells, CEACAM1 is targeted by pathogens such as *Neisseria meningitidis*, *Neisseria gonorrhoeae* [26], *Haemophilus influenzae* [27], and *Moraxella catarrhalis* [28], [29] via one or more adhesins on their surface to alter the target cell function. Whether such bacterial CEACAM-binding proteins can induce monocyte differentiation in a receptor-dependent manner remains to be shown.

Previous studies have identified a number of bacterial virulence-associated adhesins such as the Opa proteins of *Neisseriae* [26], the protein P5 of *H. influenzae* [27]and the UspA1 protein of *M. catarrhalis* [28] that can specifically bind to the N-terminal domain of CEACAM1. Furthermore, it has been reported that UspA1 protein of *M. catarrhalis* induces CEACAM1-dependent apoptosis in alveolar epithelial cells and that this might contribute to the pathogenesis of chronic obstructive pulmonary disease (COPD) [30]. In addition, UspA1 also helps *M. catarrhalis* evade host immunity through inhibiting both the alternative and classical pathways of the complement system [31]. It has also been reported that *M. catarrhalis* infected alveolar epithelium induced monocyte recruitment [32], but little is known about the potential effects of *M. catarrhalis* on the recruited monocyte differentiation after *M. catarrhalis* infection. While monocytes may encounter multiple stimulants presented on the bacterial surface (such as UspA1, LPS), engagement of UspA1 with CEACAM1 has been reported to be involved in the regulation not only of epithelial function (as described above) but also of T cell function upon CEACAM-1 cross-linking [33]. Therefore, in this study, we focused on addressing the potential effects of the CEACAM1 ligand UspA1 (and in particular the recombinant UspA1 fragment rD-7 that binds to CEACAM1 [34], [35]) on monocyte function. It is also noteworthy that UspA1 has been deemed a potential vaccine antigen to combat *M. catarrhalis* infections [34], [36], [37], [38] and as such it may be administered in its purified form either as a whole molecule or as the rD-7 fragment. This might induce bactericidal antibodies and/or inhibit bacterial colonization by binding to epithelial CEACAMs [34]; however, these components may also affect immune function on encountering CEACAMs on immune cells. A fuller knowledge of how such *M. catarrhalis* components affect the human immune response is therefore of particular interest.

In addition to the recombinant molecules such as rD-7 corresponding to the CEACAM1-binding region of UspA1, we have generated a control molecule r6–8 (based on a sequence of UspA1 distinct from its receptor-binding sequence) and rD-7/D (a mutant form of rD-7 with diminished ability to bind to CEACAM1) [34], [35]. Using these tools, we have investigated their effects on differentiation of and cytokine secretion by human myeloid-derived CD14+ monocytes.

## Materials and Methods

### Ethics Statement

Buffy Coats were obtained from healthy human volunteers (National Blood Service, Bristol, UK; Shandong Blood Center, Jinan, China) and written approval was obtained in each case. The collection and use of blood and the research described complies with the relevant guidelines and institutional practices (University of Bristol Hospital Trust Local Research Ethical Committee E4388) and also complies with relevant guidelines and institutional practices from the Ethics Committees of Qilu Hospital of Shandong University. Our study was specially approved by University of Bristol Hospital Trust Local Research Ethical Committee and the Ethics Committees of Qilu Hospital of Shandong University (No.1179).

### Antibodies, Cytokines, and Reagents used are Listed Below

Rabbit anti-human Carcinoembryonic Antigen polyclonal antibody (Ab) (Dako, Denmark Ab A0115); rabbit IgG control (Vector, CA, USA); PE-conjugated anti-CEACAM1 monoclonal antibody (mAb) (R and D Systems MAb2244); FITC-conjugated mouse anti-human CD14 mAb, human FcR Blocking Reagent, CD14+/CD15+ Microbeads and LS columns (Miltenyi biotec, Germany); PE-conjugated mouse anti-human CD86 mAb and PE-Cy7-conjugated mouse anti-human CD80 mAb (Biolegend, CA, USA); PE-conjugated mouse anti-human CD206 mAb and FITC-conjugated mouse anti-human CD15 mAb (Becton Dickinson, CA, USA); PE-conjugated goat anti-rabbit IgG antibody, endotoxin-free water and Histopaque-1077 (Sigma, USA); fixable Vital dye eFluor 780 (ebioscience, CA, USA); LPS-EK Ultrapure (Invivogen, CA, USA); Ni-NTA agarose and polypropylene column (QIAGEN); Pierce BCA Protein Assay Kit and Pierce LAL Chromogenic Endotoxin Quantitation Kit (Thermo Scientific); Proteome Profiler kits (R&D systems, UK). Antiserum to recombinant rD-7 was raised in mice and has been described previously [29].

### Production and Identification of Recombinant rD-7, rD-7/D and r6–8 Proteins

The recombinant UspA1 fragment rD-7 represents amino acids 527–665 of UspA1. The protein rD-7/D corresponds to the same region of UspA1 (527–665) but with two point mutations introduced by site directed mutagenesis (M568A and A588Q) of full length UspA1. The recombinant protein r6–8 corresponds to amino acids 659–863 of UspA1. Unlike rD-7, neither r6–8 nor rD-7/D binds to CEACAM1. All three proteins were produced using the pQE30 expression system (Qiagen) and have been described previously [35]. Purified proteins were detected using an anti-His-tag antibody and in the case of rD-7, also a soluble CEACAM1-Fc construct [34] by western blotting (Fig. S1). The endotoxin levels in all purified proteins were less than 1.0 EU/µg as determined by Limulus Amoebocyte Lysate method.

### Western Blot

The concentrations of rD-7 and rD-7/D were detected by BCA Protein Assay Kit (Pierce) and the samples were heated for 5 min at 100°C before loading 1 µg protein per well from each sample on to a 12.5% gel. After separation by electrophoresis, the bands of the expected size (17 kDa) were observed in Coomassie Blue stained gels. And proteins were transferred to a nitrocellulose membrane (Millipore) and the bands of expected size (17 kDa) were observed in Western blots detected with anti-His tag antibody (Novagen) and CEACAM1-Fc construct described previously [39]. rD-7 and rD-7/D were detected with anti-His tag antibody followed by anti-mouse IgG secondary antibody conjugated to alkaline phosphatase (Sigma). CEACAM1-Fc bound to rD-7 but not rD-7/D. Receptor binding was detected using an anti-human-Fc antibody conjugated to alkaline phosphatase. In the case of both anti-His tag and receptor overlay, alkaline phosphatase conjugates binding was detected by the addition of nitroblue tetrazolium and 5-bromo-4-chloro-3-indoyl phosphate as substrates (Sigma).

### Digestion of rD-7 and rD-7/D

Samples of the purified recombinant molecules were digested using 200 µg/ml Proteinase K (PK) at 37°C for 3 h and the digested samples heated at 95°C for 1 h to inactive the enzyme. The absence of intact proteins in the relevant samples was confirmed using SDS-PAGE.

### Isolation of Peripheral Blood Cells

PBMC were isolated from healthy human peripheral blood derived buffy coats using Histopaque-1077. CD14+ monocytes or CD15+ cells (human eosinophils and neutrophils) were then separated by magnetic cell sorting using human CD14 and CD15 MicroBeads (Miltenyi Biotech) according to the manufacturer’s instructions. The purity of the two subsets of cells was greater than 95% as determined using anti-CD14 or anti-CD15 mAbs.

### Monocyte Activation

Freshly isolated monocytes (1×10^6^) were cultured in complete RPMI medium containing 2 mM L-glutamine, 25 mM HEPES, 100 U/ml penicillin, 100 µg/ml streptomycin and 10% human serum of blood group AB (Sigma) in the absence or presence of 2.5 µg/ml of the recombinant proteins rD-7, r6–8 or rD-7/D, or with 2 µg/ml of LPS in 24-well plates. After 24 h, cells were harvested and analysed by flow cytometry and culture supernatants were collected and stored at −80°C for cytokine detection. To block the binding of rD-7 to CEACAM1 in some experiments, monocytes were treated with A0115 or IgG control (50 µg/ml) for 1 h prior to the addition of the recombinant molecules as above. After 24 h, cells were collected and surface staining conducted as described below.

### Flow Cytometry

Monocytes were harvested, washed with phosphate buffered saline (PBS), and resuspended in FACS buffer (2% human serum, 0.5 mM EDTA in PBS). After staining with the fixable vital dye eFluor 780, the cells were treated with FcR blocking buffer containing 5% human serum for 15 min at 4°C. For detection of surface receptors, cells were then treated with PE-conjugated antibodies against CD86 or CD206; FITC-conjugated anti-CD14; PE-Cy7 conjugated CD80; rabbit anti-CEACAM Ab A0115 and PE-conjugated anti-rabbit IgG or PE-conjugated anti-CEACAM1 mAb (R and D Systems MAb2244). After 30 min incubation at 4°C, the cells were washed with 1 ml FACS buffer and fixed in 200 µl 1% paraformaldehyde in PBS.

For detection of surface-bound rD-7 or rD-7/D and their inhibition by anti-CEACAM antibody, 1×10^6^ freshly isolated monocytes or CD15+ cells were first incubated in the presence of A0115 (50 µg/ml) or its IgG control (50 µg/ml) for 30 min on ice and then with the recombinant bacterial molecules (2.5 µg/ml) for 1 h on ice. After treatment, cells were incubated with the vital dye and blocking buffer as above before the addition of mouse anti rD-7 antiserum [29], followed by PE-conjugated anti mouse IgG and FITC-conjugated anti CD14 for gating monocytes and FITC-conjugated anti CD15 Abs for gating CD15+ cells. Cells were washed, fixed in 200µl 1% paraformaldehyde in PBS and analysed by flow cytometry.

### Luminex Assays

IL-6, IL-10, IL-12p70 and TNF-α in supernatants from monocytes stimulated with rD-7 (2.5 µg/ml), rD-7/D (2.5 µg/ml), or LPS (2 µg/ml) from four donors were quantitatively measured by using MILLIPLEX MAP Human Cytokine/Chemokine Magnetic Bead Panel I, Immunology Multiplex Assay according to the manufacturer’s instructions (Millipore, MA, USA). Data were analysed using the MAGPIX system (Millipore, MA, USA).

### Proteome Profiler Array

Monocytes from three donors were used for further analysis. The Proteome Profiler human cytokine array Panel A kit was used to measure the relative levels of cytokines and chemokines in cell culture supernatants after stimulation with the recombinant UspA1-derived molecules or LPS as above. The assays were performed in duplicate according to the manufacturer’s instructions (R&D Systems, UK) using 1 ml of each supernatant. Densities of dot blots were analysed using Odyssey Imaging System (LI-COR, Nebraska, USA).

### Statistical Analysis

Each experiment was performed on at least three separate occasions and the number of independent experiments carried out are stated in figure legends together with the number of replicates for each data point. Statistical analyses were performed using SPSS version 11.5. Normality of the data were tested using the Shapiro-Wilk test. Data were normally distributed, and the results are presented as the mean ± standard deviation (SD). A one-way analysis of variance (ANOVA) test was used for statistical comparisons between groups (where number of groups ≥3) and Dunnett test or Tukey test were used for post hoc analysis of the significant ANOVA results. P<0.05 was considered statistically significant.

## Results

### Modulation of Monocyte Differentiation by *M. catarrhalis* UspA1-derived Recombinant Fragment rD-7

**Figure 1:**
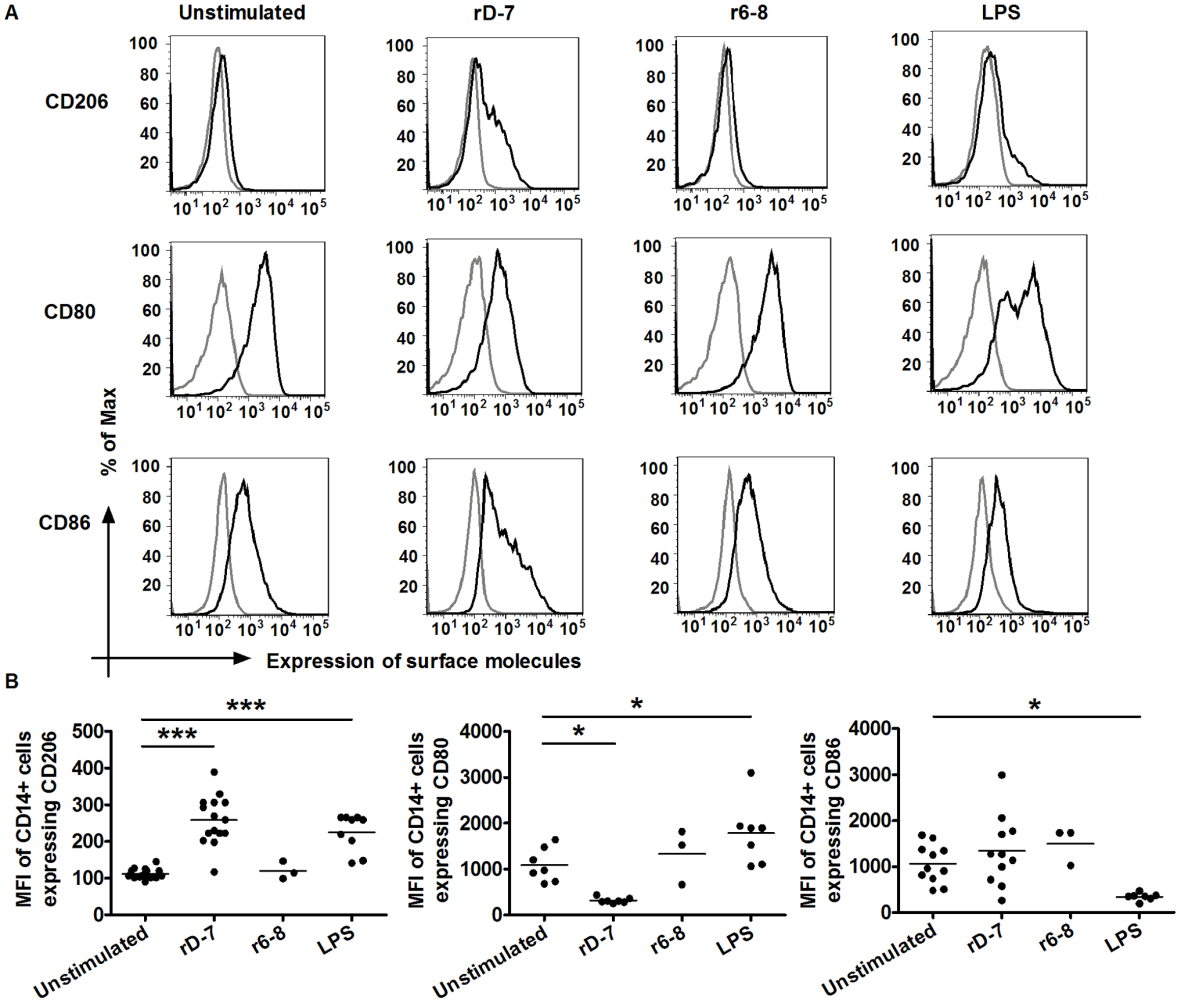
CD206, CD80 and CD86 expression on CD14+ cells after stimulation with rD-7 and r6–8. (A) 1×10^6^ monocytes were treated with rD-7 (2.5 µg/ml), r6–8 (2.5 µg/ml) or LPS (2 µg/ml) for 24 h, cells collected and live cells sorted using the vital dye eFluor 780. CD14+ cells were then gated for the analysis of CD206, CD80 and CD86 expression by flow cytometry. Black and grey histograms represent stained cells and unstained cells respectively. (B) The MFI of CD14+ cells expressing CD206, CD80 or CD86 are shown. rD-7, r6–8 or LPS was used in three independent experiments at least and each dot represents a separate experiment (*, P<0.05; ***, P<0.001).

In order to investigate the effect of *M. catarrhalis* CEACAM-binding surface adhesin UspA1 on monocyte differentiation and function, monocytes were incubated with the CEACAM-binding recombinant molecule rD-7 or a control molecule, r6–8, corresponding to a region of UspA1 not involved in CEACAM binding. After 24 h, their effects on CD206 (the alternative activation marker of M2 macrophages), CD80 and CD86 expression were analysed using flow cytometry. The mean fluorescence intensity (MFI) of CD14+ cells expressing CD206 was significantly increased by rD-7 compared with untreated or r6–8-treated cells (Fig. 1). LPS also increased the MFI of CD14+ cells expressing CD206 but to a lesser extent than rD-7. Additionally, while LPS increased CD80 expression, rD-7 decreased its expression; in contrast r6–8 did not affect CD80 expression. Neither rD-7 nor r6–8 affected the expression of CD86 by monocytes.

### The Increased MFI of CD14+ Cells Expressing CD206 Modulated by rD-7 was Dependent on the Intact Protein

**Figure 2:**
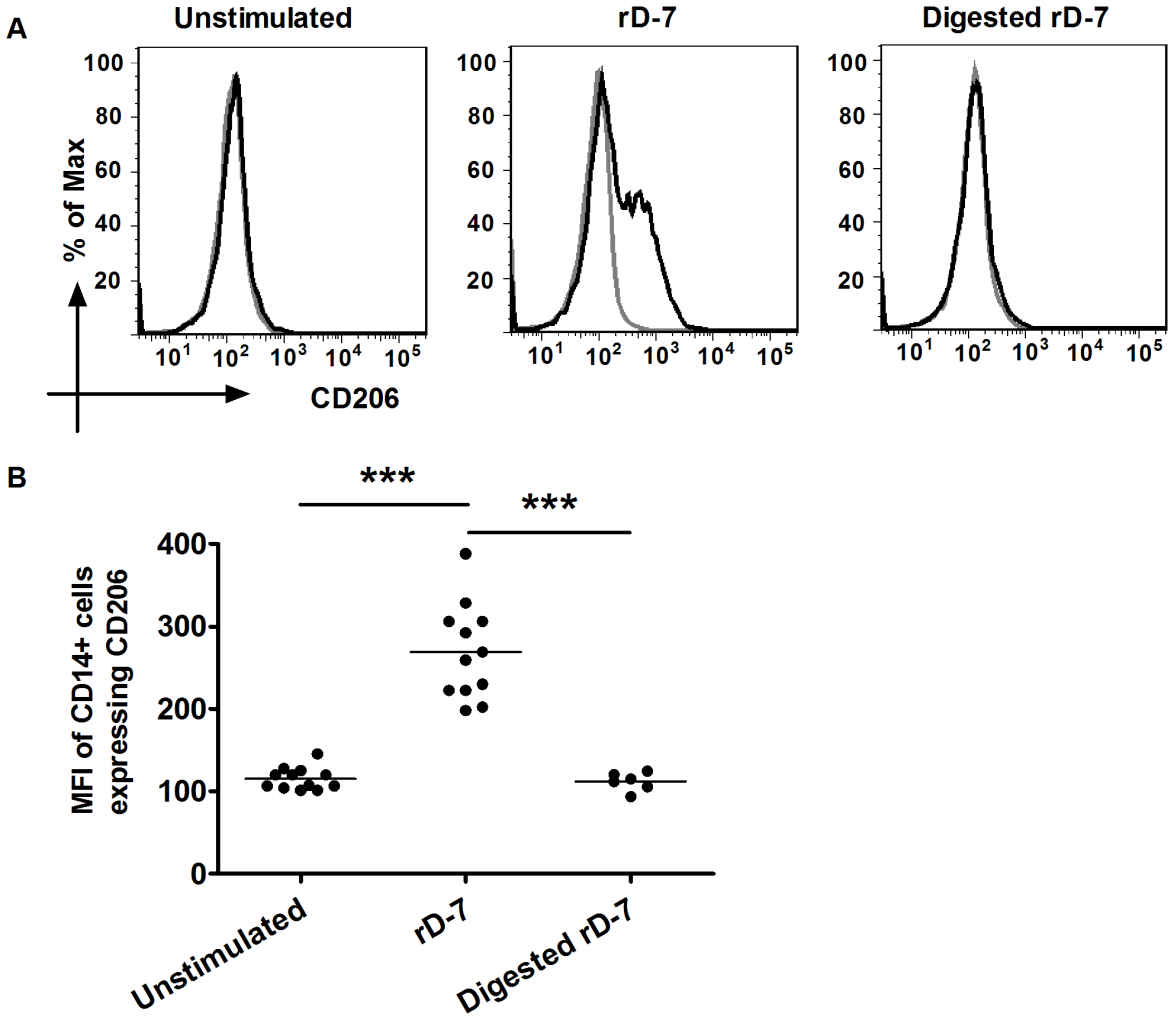
CD206 expression on CD14+ cells after stimulation with digested rD-7. (A) Monocytes were treated with rD-7 (2.5 µg/ml), or PK-digested rD-7 for 24 h and CD206 expression on CD14+ cells was analysed as described in legend to Fig. 1. Black and grey histograms represent stained cells and unstained cells respectively. (B) The MFI of CD14+ cells expressing CD206 are shown.rD-7 or digested rD-7 was used in three independent experiments at least and each dot represents a separate experiment (***, P<0.001).

As LPS also increased the MFI of CD14+ cells expressing CD206 after 24 h stimulation (Fig. 1A), we carried out further experiments to ascertain whether residual levels of endotoxin in the protein preparations were responsible for the observed effects. Samples of rD-7 were digested with Proteinase K and added to monocyte differentiation cultures. The purity and proteolytic digestion of rD-7 was confirmed by SDS-PAGE (Fig. S2). As shown in Fig. 2A, CD206 expression was increased by rD-7, whilst PK-treated rD-7 preparations lost the ability to increase CD206 expression by monocytes (Fig. 2B). These data indicate that CD206 modulation was mediated by the protein fraction within the rD-7 preparations.

### CD14+ Monocytes Express CEACAM1, the Receptor of rD-7

**Figure 3:**
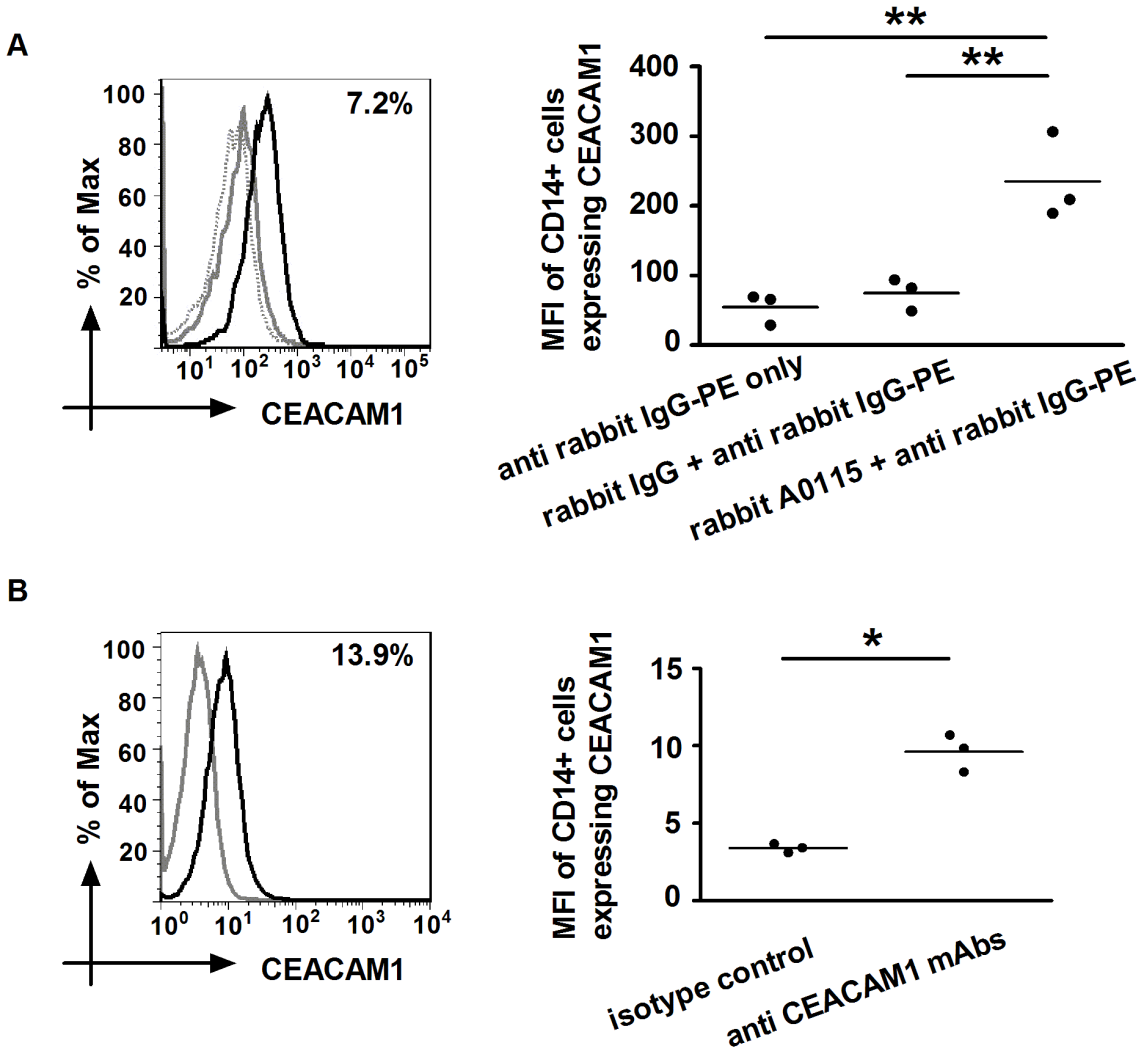
Analyses of CEACAM1 expression on CD14+ monocytes. (A) Monocytes were incubated with rabbit anti-CEACAM antibody A0115 (black histogram) or its IgG control (grey histogram) followed by secondary PE-conjugated anti-rabbit IgG and FITC-conjugated CD14 mAb. Dotted histogram represents secondary antibody only control. (B) Cells were also labelled with PE-conjugated anti-CEACAM1 mAb (R and D Systems MAb2244) (black histogram) or its isotype control (grey histogram) and FITC-conjugated CD14 mAb. Live CD14+ cells were analysed and the percentages of cells expressing CEACAM1 were found to be 7.2±5.1% using A0115 and 13.9±4.1% using MAb2244. The MFI plots of CEACAMs on CD14+ monocytes are shown on the right (*, P<0.05; **, P<0.01).The three dots in each case represent data from monocytes obtained from three donors. Note the differences in the values shown for the two antibodies could also be assigned to the experiments being performed using two different instruments (Canto II, University of Bristol and Calibur, Shandong University respectively) using different settings.

In order to determine whether the rD-7-induced modulation of CD206 expression by monocytes was CEACAM-dependent, we first determined the expression of CEACAM1 on monocytes by flow cytometry using the polyclonal antibody A0115 as well as an anti-CEACAM1 mAb (R and D Systems MAb2244) [40]. As reported previously by Yu et al [24], low numbers of monocytes expressing CEACAM1 were detected by both antibodies (Fig. 3).

### rD-7-induced CD206 Expression on Monocytes is Independent of the Ability to Bind CEACAM1

**Figure 4:**
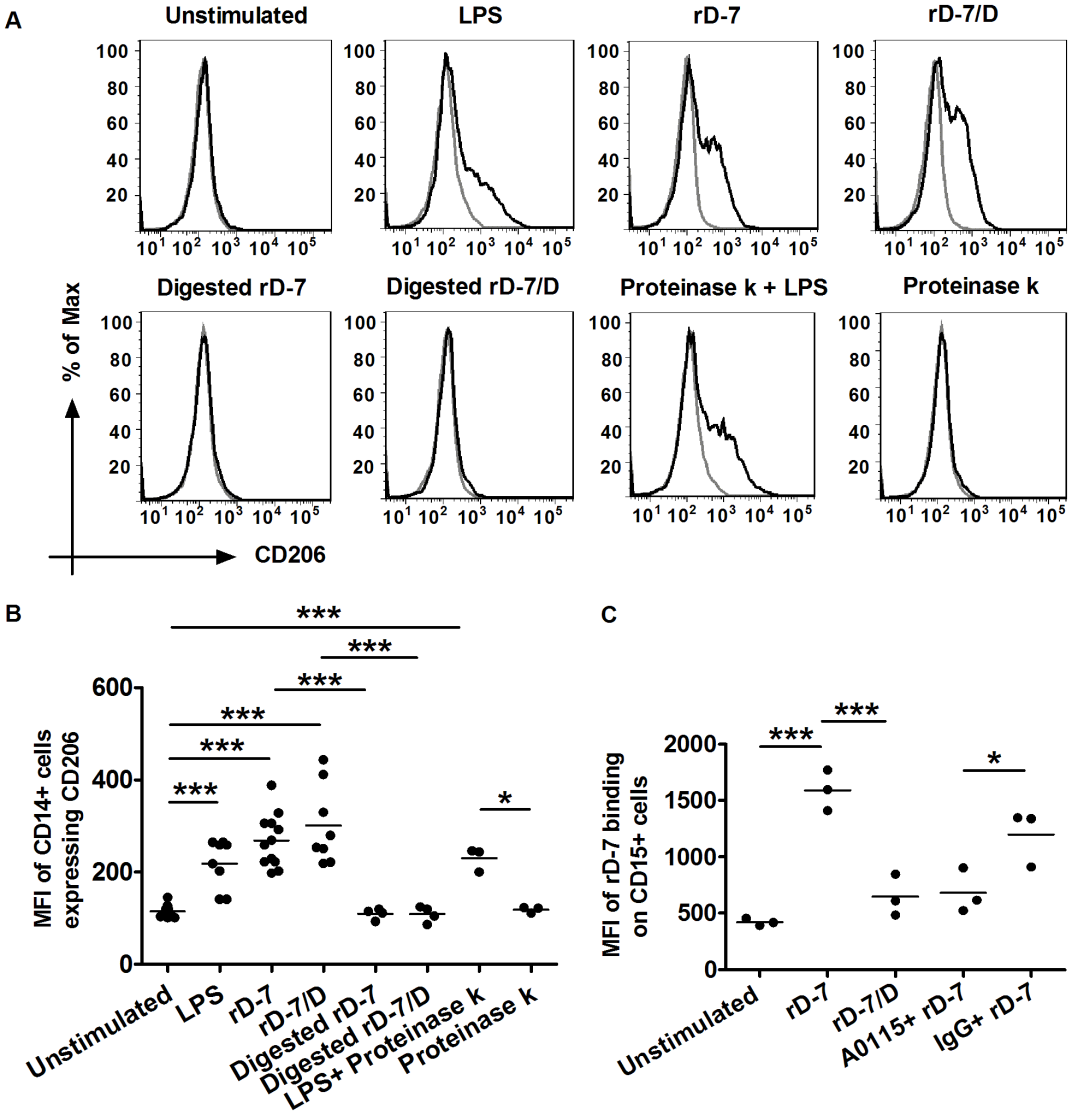
CD206 expression on CD14+ cells after stimulation with rD-7/D. (A) Monocytes treated with rD-7, rD-7/D, or PK-digested samples of the proteins (2.5 µg/ml each) were collected after 24 h, live CD14+ cells were gated and CD206 expression was analysed. Black and grey histograms represent stained and unstained cells respectively. (B) The MFI of CD14+ cells expressing CD206 are shown. rD-7, rD-7/D, LPS, digested rD-7, digested rD-7/D or Proteinase k was used in three independent experiments at least and each dot represents a separate experiment. (C) Surface binding of rD-7 to CD15+ cells. 1×10^6^ CD15+ cells were blocked with A0115 or its IgG control (50 µg/ml) for 30 min on ice, the cells were then stimulated with rD-7 or rD-7/D (2.5 µg/ml each) for 1 h on ice, rD-7 bound to live CD15+ cells was detected with primary mouse anti rD-7 antiserum and secondary PE-conjugated anti mouse IgG Abs by flow cytometry. (*, P<0. 05; **, P<0. 01; ***, P<0. 001).

In further experiments, to assess the role of CEACAM-1 in rD-7-induced CD206 expression, we used rD-7 as well as an rD-7 mutant with attenuated CEACAM binding (rD-7/D) [35]. When monocytes were stimulated with either rD-7 or rD-7/D for 24 h, CD206 expression was upregulated to similar levels in each case. No such upregulation was found using protease-treated rD-7 and rD-7/D. As observed previously, control stimulation with LPS alone also increased CD206 expression which was not altered by PK digestion (Fig. 4A and B).

For comparison, the binding of rD-7 and rD-7/D to CD15+ cells known to express high levels of CEACAMs [41] was also determined by flow cytometry. In contrast to monocytes, while rD-7 bound to CD15+ cells, rD-7/D did not bind to these cells (Fig. 4C). The specificity of rD-7 binding to CEACAMs on CD15+ cells was also determined by the use of A0115, known to block CEACAM binding by the recombinant molecule [28]. A0115 but not control IgG diminished the binding of rD-7 confirming the specificity of CEACAM binding by rD-7 on CD15+ cells (Fig. 4C).

### Confirmation of CEACAM-independent Binding and Stimulation of CD206 Expression on CD14+ Cells by rD-7 and rD-7/D by Antibody Inhibition Assays

**Figure 5:**
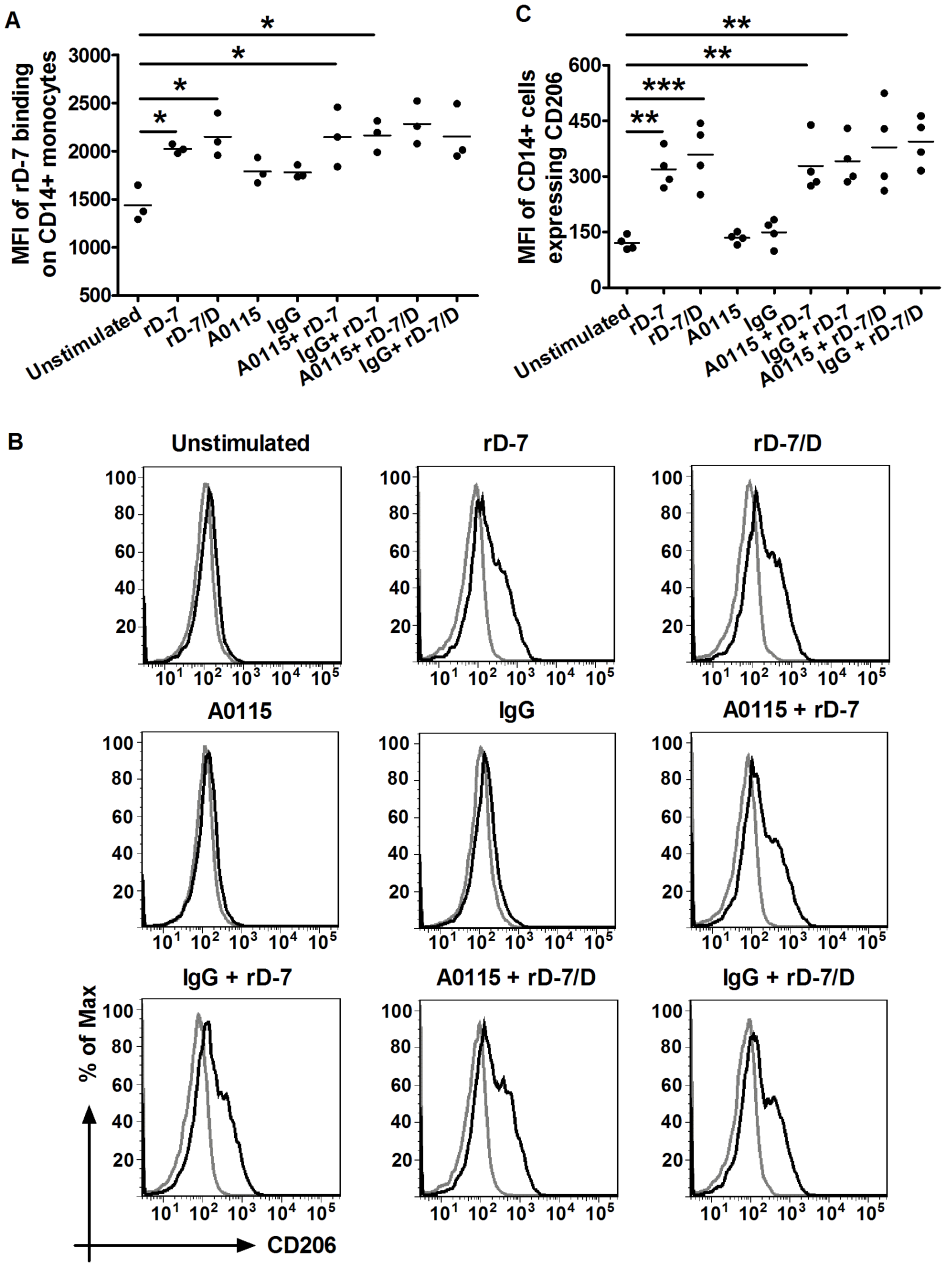
Ab A0115 does not affect binding of rD-7 or CD206 expression on CD14+ cells. (A) Monocytes were pre-treated with A0115 or its IgG control (50 µg/ml each) for 30 min on ice and then rD-7 (2.5 µg/ml) or rD-7/D (2.5 µg/ml) added for 1 h. The surface bound of rD-7 on live CD14+ cells was detected with primary mouse anti rD-7 antiserum and secondary PE-conjugated anti mouse IgG Abs and FITC-conjugated anti CD14 mAb. (B) Monocytes were treated with A0115 or its IgG control (50 µg/ml) for 1 h and the cells were then stimulated with rD-7 or rD-7/D (2.5 µg/ml each) for 23 h. CD206 expression was analysed on live CD14+ cells. Black and grey histograms represent stained cells and unstained cells. (C) The MFI of CD14+ cells expressing CD206 are shown. Experiments were conducted three and four times as shown. Each dot represents an independent experiment. (*, P<0. 05; **, P<0. 01; ***, P<0. 001).

As rD-7 binding to CD15+ cells could be readily inhibited by A0115, this antibody was used to first assess its effect on binding of the recombinant molecule to CD14+ cells by flow cytometry. While rD-7 (and rD-7/D also tested in parallel) bound to the surface of monocytes, A0115 did not block the binding as observed with IgG control (Fig. 5A). This was in contrast to results observed with rD-7 binding to CD15+ cells (Fig. 4C).

Together, the above data suggested strongly that rD-7 may modulate monocyte differentiation in a CEACAM-independent manner. This was further confirmed by treating freshly isolated monocytes with A0115 before stimulation with rD-7 or rD-7/D for 24 h. Both rD-7 and rD-7/D enhanced CD206 expression and this remained unaffected in the presence of A0115 (Fig. 5B) again indicating that monocyte differentiation modulated by either rD-7 or rD-7/D was not dependent on their interaction with CEACAMs.

### IL-6, TNF-α, IL-10 and IL-12p70 Secretion from Monocytes Stimulated by rD-7 and rD-7/D

**Figure 6:**
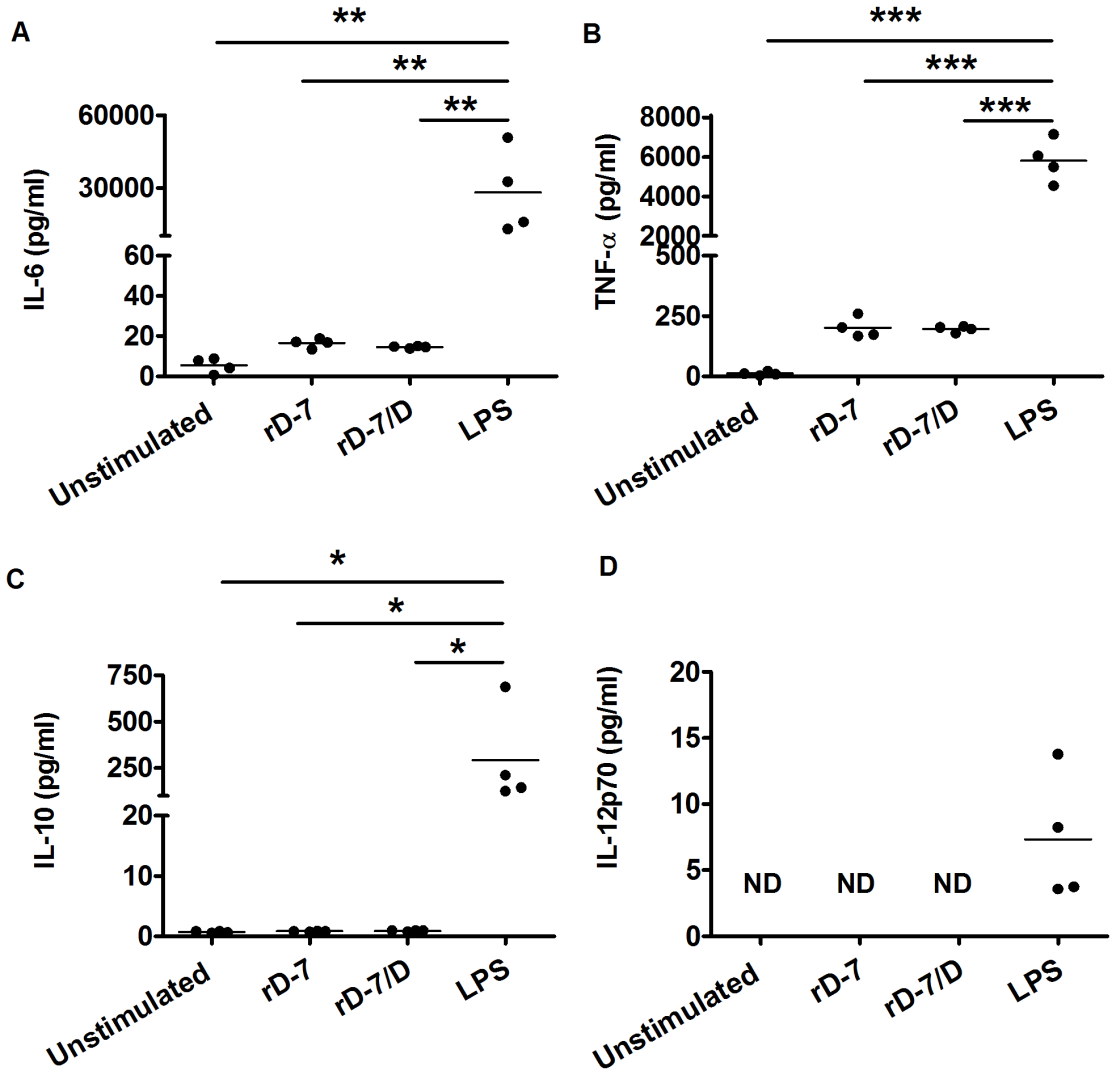
TNF-α, IL-6, IL-10 and IL-12p70 from monocytes modulated by rD-7 or rD-7/D. Supernatants of 1×10^6^ monocytes stimulated with rD-7 (2.5 µg/ml), rD-7/D (2.5 µg/ml) or LPS (2 µg/ml) for 24 h were collected and TNF-α (A), IL-6 (B), IL-10 (C) and IL-12p70 (D) secretion was determined by using Luminex assay kit. Each dot represents the mean of triplicate estimations within an experiment and the four dots in each case represent data from monocytes obtained from four donors (*, P<0.05; **, P<0.01, ***, P<0.001).

One important function of monocytes and macrophages is to produce pro-inflammatory and anti-inflammatory cytokines, such as IL-12, TNF-α, IL-6 and IL-10, which may shape the nature of the adaptive immune response. Therefore, IL-10, IL-12p70, IL-6 and TNF-α were quantified by Luminex assays, using monocyte supernatants from four donors (Fig. 6). The results confirmed that rD-7 and rD-7/D did not increase the secretion of IL-10 or IL-12p70, whilst neither rD-7 nor rD-7/D induced IL-6 and TNF-α secretion compared with the unstimulated group from the four donors.

### Cytokines and Chemokine Expression Profiles from Monocytes Modulated by rD-7 or rD-7/D

**Figure 7:**
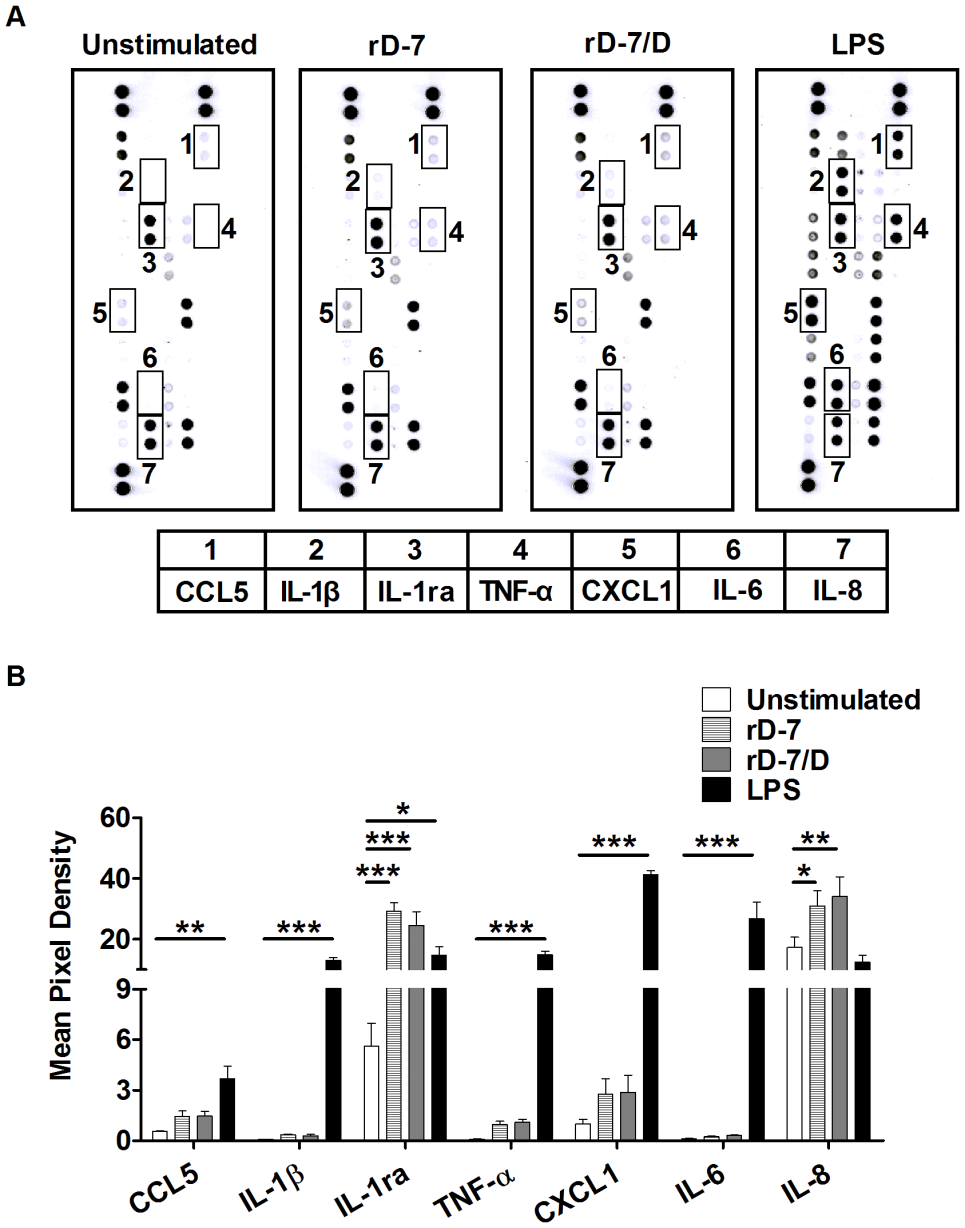
Cytokine and chemokine expression profiles from monocytes modulated by rD-7. Supernatants of monocytes stimulated with rD-7 or rD-7/D from three donors were used to assess their cytokine and chemokine expression using Proteome Profiler R&D Systems according to the manufacturer’s instructions. The experiments were performed in duplicate using monocytes from three of the four donor samples shown in Fig. 6. Results of one of three donor samples are shown in A and the rest are shown in Fig. S3 (*, P<0.05; **, P<0.01, ***, P<0.001). The mean pixel density of dots shown in B were obtained using Odyssey Imaging System using data from all three donors.

As above assays using several donors gave similar results for rD-7 and rD-7/D (similar effects on the expression of CD206, CD80, CD86 and on the secretion of IL-10, IL-12p70, IL-6, TNF-α), three representative supernatants from the four donors used in Luminex assays were further used to determine the broader cytokine and chemokine expression profiles as assessed by proteome profiler assay. We observed a significant increase in IL-1ra secretion produced by monocytes stimulated with rD-7 or rD-7/D for 24 h. Interestingly, the quantity of IL-1ra released following treatment with rD-7 or rD-7/D was similar to that from the LPS-stimulated group (Fig. 7). In addition, IL-8 secretion was also increased by either rD-7 or rD-7/D when compared to the unstimulated group (Fig. 7). IL-2, IL-4, IL-5, IL-10, IL-12p70, IL-17, IL-32a, MCP-1, MIP-1β, CXCL10 and CXCL12 were undetectable in rD-7- or rD-7/D-stimulated groups (Mean pixel density was less than 0.1). There was no significant change for C5a, CD40 ligand, G-CSF, GM-CSF, CXCL1, CCL1, sICAM-1, IFN-γ, IL-1α, IL-1β, IL-6, IL-13, IL-16, IL-17E, IL-23, IL-27, CXCL11, MIF, CCL3, Serpin E1, CCL5, TNF-α, sTREM-1 secretion between the rD-7 or rD-7/D groups and the unstimulated group (Fig. 7).

## Discussion

During the microbial infection process, circulating blood monocytes migrate from the vasculature into the extra-vascular compartment where they mature into tissue macrophages [42]. In 1992, Stahl reported that the mannose receptor CD206 was not found on circulating monocytes, while it was abundantly expressed on differentiated macrophages [43] and the LPS-induced differentiation of monocytes to macrophages involved NFκB [42]. In our studies, we also observed that the expression of CD206 on unstimulated CD14+ monocytes was low but its expression on CD14+ cells treated with rD-7 for 24 h was significantly increased, suggesting rD-7 initiates monocyte differentiation into macrophages. In terms of classically activated M1 macrophages and alternatively activated M2 macrophages, it has been shown that monocytes/macrophages co-cultured with Tregs display typical features of alternative activated macrophages, including up-regulated expression of CD206 and CD163, an increased production of CCL18, and an enhanced phagocytic capacity [44]. Enhanced CD206 may be involved in phagocytosis, antigen processing and presentation, cell migration and intracellular signalling [45], [46].

CD80 is expressed on antigen-presenting cells. It provides a co-stimulatory signal necessary for T cell activation and survival, and is the ligand for CD28 and CTLA-4 (CD152) [47], [48], which have crucial yet opposing functions in T-cell stimulation. CD80 works in tandem with CD86 in priming T cells and it is also considered a marker of DC and macrophage maturation [49], [50]. Notably, we found that CD80 expression on CD14+ cells was significantly decreased by rD-7 after 24 h stimulation, suggesting that despite driving differentiation, rD-7 may impair the acquisition of full APC function. This property of rD-7 was in marked contrast to the effects of LPS, which increased CD80 expression on CD14+ cells. Similarly, rD-7 did not alter CD86 expression on CD14+ cells in our experiments, whereas as is consistent with other reports, LPS induced early upregulation of CD86, but then levels declined such that by 24 h, levels of CD86 expression on LPS-treated monocytes were lower than on untreated controls [51]. These data suggest that the effects of LPS and rD-7 on monocyte differentiation were different, and may point towards modulation of the differentiation pathway away from a fully activated phenotype.

It has been reported that the UspA1 protein of *M. catarrhalis* induces CEACAM1-dependent apoptosis in alveolar epithelial cells and that this might contribute to the pathogenesis of chronic obstructive pulmonary disease (COPD) [30]. Besides, the UspA1 protein may also modulate the function of other cells expressing CEACAM1, including migrated CEACAM1 expressing monocytes and activated T cells at inflammatory sites. Using primers based on the UspA1 gene sequence of *M. catarrhalis*, recombinant polypeptides of different length were generated. rD-7 represents residues 527–677, and rD-7/D represents residues 527–677 but with two mutations, M568A and A588Q, whilst r6–8 represents residues 659–863 [34]. We observed that monocyte differentiation was modulated by rD-7 and rD-7/D but not r6–8, implying the receptor target responsible for monocyte differentiation by rD-7 and rD-7/D was contained within residues 527–677 of UspA1.

We have determined the cytokine secretion profiles after stimulation with rD-7. There were marked differences in the profiles of cytokines induced in response to rD-7 when compared to LPS. rD-7 appeared to induce a response involving primarily IL-1ra, an inhibitor of the pro-inflammatory effects of IL-1α and IL-1β [52], along with low levels of pro-inflammatory factors, TNF-α, IL-1β and IL-6. In contrast, LPS induced far higher levels of pro-inflammatory TNF-α, IL-1β and IL-6. Interleukin-8 was produced at high levels by the cells in the absence of stimulation, levels increasing to a small extent further following rD-7 exposure. The rD-7 and LPS induced cytokine profiles were consistent in both assays used to assess their production and suggest that rD-7 modulates monocyte activation in a way that is distinct from LPS. Despite the continued presence of IL-8 production, the overall profile induced by rD-7 in which IL-1ra is stimulated in the relative absence of pro-inflammatory IL-1β, TNF-α and IL-6 is suggestive that the protein may regulate rather than overtly drive inflammation.

CEACAM1 is expressed on several types of cell, serving as a sophisticated and multi-potent signalling molecule which participates in regulating a variety of important cellular activities. Human monoblast cell lines U-937 and THP-1 have been shown to express CEACAM1 at the gene and protein level [53], and CEACAM1 was found to be expressed by primary CD14+ monocytes, albeit to a lower level than lymphocytes [24]. We also found CEACAM expression to be low on CD14+ monocytes using anti-CEACAM antibodies. According to the type and number of amino acid residues in the cytoplasmic domain, CEACAM1 is classified into CEACAM1-Long (73 amino acids) and CEACAM1-Short isoforms (10 amino acids). The CEACAM1-L contains two intracellular ITIMs like motifs not present in CEACAM1-S [54]. Activation of CEACAM1-L leads to the recruitment of Src homology 2 domain containing protein tyrosine phosphatase-1 (SHP-1) and SHP-2 to phosphorylated ITIM [55], [56], which initiates a number of signalling pathways. RT-PCR has been performed to ascertain that CEACAM1-3L (3 extracellular domains, 207 bp product) and CEACAM1-4L (4 extracellular domains, 506 bp product) were co-expressed by primary human monocytes [24]. We aimed to investigate whether the differentiation induced by rD-7 was mediated in a CEACAM1-dependent manner. For this reason, a control protein rD-7/D that displayed greatly reduced binding to CEACAM1 was used for stimulation. Unexpectedly, rD-7/D also increased CD206 expression on monocytes and there was no significant difference between CD206 expression and cytokine secretion in the rD-7 and rD-7/D treated cells, suggesting that the effects of rD-7 on monocytes differentiation were not mediated in a CEACAM-dependent manner. To test this hypothesis, we used the antibody A0115 to block the binding of rD-7 to CEACAMs [28]. The antibody neither affected the surface binding of rD-7 or rD-7/D nor blocked the increase in CD206 expression. In addition, using CD15+ neutrophils expressing high levels of CEACAM1, we found little binding of rD-7/D while rD-7 bound to the cells in a CEACAM-dependent manner which was inhibited by A0115. Although our data suggest that there is predominantly a CEACAM-independent mechanism for rD-7 binding to monocytes, it is also feasible that it may bind to a lesser extent in a CEACAM-dependent manner. However, only a small number of monocytes (∼5%) have been reported to express CEACAMs [24] in contrast to CD15+ neutrophils (∼100%) [41]. The monocyte receptor/s targeted by the region of *M. catarrhalis* UspA1 represented specifically in rD-7 and rD-7/D remain unknown and require further investigation.

In summary, our studies have shown that *M. catarrhalis* UspA1-derived recombinant molecules rD-7 and rD-7/D induced monocytes differentiation into a CD14+CD206+ phenotype. The expression of CD80 was significantly decreased by rD-7, and high levels of IL-1ra secretion was induced. Taken together with the observation that of the pro-inflammatory cytokines tested, IL-8 was the only one where levels remained high after rD-7 treatment, our findings suggest that it may regulate rather than induce inflammation. Both rD-7 and rD7/D bound to and modulated monocyte function in a CEACAM1-independent manner. The nature of this receptor has yet to be elucidated and remains an exciting target for future research. Further understanding of the mechanisms via which *M. catarrhalis* may modulate monocyte function via its surface-expressed virulence determinants such as UspA1 will add to our knowledge and better inform future vaccine design.

## Supporting Information

Figure S1 **Purity of rD-7 and rD-7/D and their binding to soluble CEACAM1 construct.** Purified recombinant molecules were analysed by SDS-PAGE and western blotting. The bands of the expected size (17 kDa) were observed in Coomassie Blue stained gels (left) and in Western blots detected with anti-His tag antibody (middle) and CEACAM1-Fc construct described previously [39]. The 17 kDa band in the rD-7 and rD-7/D were detected with anti-His tag antibody followed by anti-mouse IgG secondary antibody conjugated to alkaline phosphatase. CEACAM1-Fc bound to rD-7 but not rD-7/D. Receptor binding was detected using an anti-human-Fc antibody conjugated to alkaline phosphatase. In the case of both anti-His tag and receptor overlay, alkaline phosphatase conjugates binding was detected by the addition of nitroblue tetrazolium and 5-bromo-4-chloro-3-indoyl phosphate as substrates.
(TIF)

Figure S2 **Proteinase K treatment of rD-7 and rD-7/D.** Purified samples of rD-7, rD-7/D, or LPS were digested by 200 µg/ml Proteinase K at 37°C for 3 h. The digested samples were heat-inactivated at 95°C for 1 h to inactive Proteinase K and analysed by SDS-PAGE. Sample 1, rD-7; Sample 2, Digested rD-7; Sample 3, rD-7/D; Sample 4, Digested rD-7/D; Samples 5 and 6, Proteinase K; Sample 7, rD-7 with heat-inactivated Proteinase K; Sample 8, rD-7/D with heat-inactivated Proteinase K.
(TIF)

Figure S3 **Cytokine and chemokine expression profiles from monocytes modulated by rD-7.** Supernatants of monocytes stimulated with rD-7 or rD-7/D from donor 2(A) and donor 3 (B) were used to assess their cytokine and chemokine expression using Proteome Profiler R&D Systems according to the manufacturer’s instructions (data supplementary to Fig. 7).
(TIF)

## References

[1] Geissmann F, Manz MG, Jung S, Sieweke MH, Merad M, et al (2010) Development of monocytes, macrophages, and dendritic cells. Science 327: 656–661. 20133564 10.1126/science.1178331 PMC2887389

[2] Hoebe K, Janssen E, Beutler B (2004) The interface between innate and adaptive immunity. Nat Immunol 5: 971–974. 15454919 10.1038/ni1004-971

[3] Tacke F, Randolph GJ (2006) Migratory fate and differentiation of blood monocyte subsets. Immunobiology 211: 609–618. 16920499 10.1016/j.imbio.2006.05.025

[4] Dominguez PM, Ardavin C (2010) Differentiation and function of mouse monocyte-derived dendritic cells in steady state and inflammation. Immunol Rev 234: 90–104. 20193014 10.1111/j.0105-2896.2009.00876.x

[5] Delneste Y, Charbonnier P, Herbault N, Magistrelli G, Caron G, et al (2003) Interferon-gamma switches monocyte differentiation from dendritic cells to macrophages. Blood 101: 143–150. 12393446 10.1182/blood-2002-04-1164

[6] Benoit M, Desnues B, Mege JL (2008) Macrophage polarization in bacterial infections. J Immunol 181: 3733–3739. 18768823 10.4049/jimmunol.181.6.3733

[7] Hoffmann R, van Erp K, Trulzsch K, Heesemann J (2004) Transcriptional responses of murine macrophages to infection with Yersinia enterocolitica. Cell Microbiol 6: 377–390. 15009029 10.1111/j.1462-5822.2004.00365.x

[8] Brubaker RR (2003) Interleukin-10 and inhibition of innate immunity to Yersiniae: roles of Yops and LcrV (V antigen). Infect Immun 71: 3673–3681. 12819047 10.1128/IAI.71.7.3673-3681.2003 PMC162007

[9] Gordon S (2003) Alternative activation of macrophages. Nat Rev Immunol 3: 23–35. 12511873 10.1038/nri978

[10] Gordon S, Martinez FO (2010) Alternative activation of macrophages: mechanism and functions. Immunity 32: 593–604. 20510870 10.1016/j.immuni.2010.05.007

[11] Kerrigan AM, Brown GD (2009) C-type lectins and phagocytosis. Immunobiology 214: 562–575. 19261355 10.1016/j.imbio.2008.11.003 PMC2702671

[12] Fleischer J, Soeth E, Reiling N, Grage-Griebenow E, Flad HD, et al (1996) Differential expression and function of CD80 (B7–1) and CD86 (B7–2) on human peripheral blood monocytes. Immunology 89: 592–598. 9014827 10.1046/j.1365-2567.1996.d01-785.x PMC1456589

[13] Wang Y, Wang H, Piper MG, McMaken S, Mo X, et al (2010) sRAGE induces human monocyte survival and differentiation. J Immunol 185: 1822–1835. 20574008 10.4049/jimmunol.0903398 PMC3671884

[14] Bogoevska V, Horst A, Klampe B, Lucka L, Wagener C, et al (2006) CEACAM1, an adhesion molecule of human granulocytes, is fucosylated by fucosyltransferase IX and interacts with DC-SIGN of dendritic cells via Lewis x residues. Glycobiology 16: 197–209. 16282604 10.1093/glycob/cwj057

[15] Nouvion AL, Beauchemin N (2009) [CEACAM1 as a central modulator of metabolism, tumor progression, angiogenesis and immunity]. Med Sci (Paris) 25: 247–252. 19361387 10.1051/medsci/2009253247

[16] Horst AK, Bickert T, Brewig N, Ludewig P, van Rooijen N, et al (2009) CEACAM1+ myeloid cells control angiogenesis in inflammation. Blood 113: 6726–6736. 19273835 10.1182/blood-2008-10-184556

[17] Zhou CJ, Qu X, Yang YM, Wang FF, Dong ZQ, et al (2009) CEACAM1 distribution and it’s effects on angiogenesis and lymphangiogenesis in oral carcinoma. Oral Oncol 45: 883–886. 19442569 10.1016/j.oraloncology.2009.03.002

[18] Gerstel D, Wegwitz F, Jannasch K, Ludewig P, Scheike K, et al (2011) CEACAM1 creates a pro-angiogenic tumor microenvironment that supports tumor vessel maturation. Oncogene 30: 4275–4288. 21532628 10.1038/onc.2011.146

[19] Boulton IC, Gray-Owen SD (2002) Neisserial binding to CEACAM1 arrests the activation and proliferation of CD4+ T lymphocytes. Nat Immunol 3: 229–236. 11850628 10.1038/ni769

[20] Lobo EO, Zhang Z, Shively JE (2009) Pivotal advance: CEACAM1 is a negative coreceptor for the B cell receptor and promotes CD19-mediated adhesion of B cells in a PI3K-dependent manner. J Leukoc Biol 86: 205–218. 19454653 10.1189/jlb.0109037 PMC2726766

[21] Skubitz KM, Campbell KD, Skubitz AP (1996) CD66a, CD66b, CD66c, and CD66d each independently stimulate neutrophils. J Leukoc Biol 60: 106–117. 8699114 10.1002/jlb.60.1.106

[22] Kammerer R, Stober D, Singer BB, Obrink B, Reimann J (2001) Carcinoembryonic antigen-related cell adhesion molecule 1 on murine dendritic cells is a potent regulator of T cell stimulation. J Immunol 166: 6537–6544. 11359805 10.4049/jimmunol.166.11.6537

[23] Huang J, Hardy JD, Sun Y, Shively JE (1999) Essential role of biliary glycoprotein (CD66a) in morphogenesis of the human mammary epithelial cell line MCF10F. J Cell Sci 112 (Pt 23): 4193–4205. 10.1242/jcs.112.23.4193 10564638

[24] Yu Q, Chow EM, Wong H, Gu J, Mandelboim O, et al (2006) CEACAM1 (CD66a) promotes human monocyte survival via a phosphatidylinositol 3-kinase- and AKT-dependent pathway. J Biol Chem 281: 39179–39193. 17071610 10.1074/jbc.M608864200

[25] Thompson JA, Grunert F, Zimmermann W (1991) Carcinoembryonic antigen gene family: molecular biology and clinical perspectives. J Clin Lab Anal 5: 344–366. 1941355 10.1002/jcla.1860050510

[26] Virji M, Watt SM, Barker S, Makepeace K, Doyonnas R (1996) The N-domain of the human CD66a adhesion molecule is a target for Opa proteins of Neisseria meningitidis and Neisseria gonorrhoeae. Mol Microbiol 22: 929–939. 8971714 10.1046/j.1365-2958.1996.01548.x

[27] Hill DJ, Toleman MA, Evans DJ, Villullas S, Van Alphen L, et al (2001) The variable P5 proteins of typeable and non-typeable Haemophilus influenzae target human CEACAM1. Mol Microbiol 39: 850–862. 11251807 10.1046/j.1365-2958.2001.02233.x

[28] Hill DJ, Virji M (2003) A novel cell-binding mechanism of Moraxella catarrhalis ubiquitous surface protein UspA: specific targeting of the N-domain of carcinoembryonic antigen-related cell adhesion molecules by UspA1. Mol Microbiol 48: 117–129. 12657049 10.1046/j.1365-2958.2003.03433.x

[29] Hill DJ, Whittles C, Virji M (2012) A novel group of Moraxella catarrhalis UspA proteins mediates cellular adhesion via CEACAMs and vitronectin. PLoS One 7: e45452. 23049802 10.1371/journal.pone.0045452 PMC3458076

[30] N’Guessan PD, Vigelahn M, Bachmann S, Zabel S, Opitz B, et al (2007) The UspA1 protein of Moraxella catarrhalis induces CEACAM-1-dependent apoptosis in alveolar epithelial cells. J Infect Dis 195: 1651–1660. 17471435 10.1086/514820

[31] Hallstrom T, Nordstrom T, Tan TT, Manolov T, Lambris JD, et al (2011) Immune evasion of Moraxella catarrhalis involves ubiquitous surface protein A-dependent C3d binding. J Immunol 186: 3120–3129. 21270401 10.4049/jimmunol.1002621

[32] Rosseau S, Wiechmann K, Moderer S, Selhorst J, Mayer K, et al (2005) Moraxella catarrhalis–infected alveolar epithelium induced monocyte recruitment and oxidative burst. Am J Respir Cell Mol Biol 32: 157–166. 15557018 10.1165/rcmb.2004-0091OC

[33] Youssef AR, van der Flier M, Estevao S, Hartwig NG, van der Ley P, et al (2009) Opa+ and Opa- isolates of Neisseria meningitidis and Neisseria gonorrhoeae induce sustained proliferative responses in human CD4+ T cells. Infect Immun 77: 5170–5180. 19720754 10.1128/IAI.00355-09 PMC2772550

[34] Hill DJ, Edwards AM, Rowe HA, Virji M (2005) Carcinoembryonic antigen-related cell adhesion molecule (CEACAM)-binding recombinant polypeptide confers protection against infection by respiratory and urogenital pathogens. Mol Microbiol 55: 1515–1527. 15720557 10.1111/j.1365-2958.2005.04487.x

[35] Conners R, Hill DJ, Borodina E, Agnew C, Daniell SJ, et al (2008) The Moraxella adhesin UspA1 binds to its human CEACAM1 receptor by a deformable trimeric coiled-coil. Embo J 27: 1779–1789. 18497748 10.1038/emboj.2008.101 PMC2396876

[36] McMichael JC (2000) Vaccines for Moraxella catarrhalis. Vaccine 19 Suppl 1S101–107. 11163472 10.1016/s0264-410x(00)00287-5

[37] Meier PS, Troller R, Grivea IN, Syrogiannopoulos GA, Aebi C (2002) The outer membrane proteins UspA1 and UspA2 of Moraxella catarrhalis are highly conserved in nasopharyngeal isolates from young children. Vaccine 20: 1754–1760. 11906762 10.1016/s0264-410x(02)00030-0

[38] Riesbeck K, Tan TT, Forsgren A (2006) MID and UspA1/A2 of the human respiratory pathogen Moraxella catarrhalis, and interactions with the human host as basis for vaccine development. Acta Biochim Pol 53: 445–456. 16964325

[39] Virji M, Evans D, Hadfield A, Grunert F, Teixeira AM, et al (1999) Critical determinants of host receptor targeting by Neisseria meningitidis and Neisseria gonorrhoeae: identification of Opa adhesiotopes on the N-domain of CD66 molecules. Mol Microbiol 34: 538–551. 10564495 10.1046/j.1365-2958.1999.01620.x

[40] Rahmoun M, Moles JP, Pedretti N, Mathieu M, Fremaux I, et al (2009) Cytokine-induced CEACAM1 expression on keratinocytes is characteristic for psoriatic skin and contributes to a prolonged lifespan of neutrophils. J Invest Dermatol 129: 671–681. 18843289 10.1038/jid.2008.303

[41] van Gisbergen KP, Ludwig IS, Geijtenbeek TB, van Kooyk Y (2005) Interactions of DC-SIGN with Mac-1 and CEACAM1 regulate contact between dendritic cells and neutrophils. FEBS Lett 579: 6159–6168. 16246332 10.1016/j.febslet.2005.09.089

[42] Takashiba S, Van Dyke TE, Amar S, Murayama Y, Soskolne AW, et al (1999) Differentiation of monocytes to macrophages primes cells for lipopolysaccharide stimulation via accumulation of cytoplasmic nuclear factor kappaB. Infect Immun 67: 5573–5578. 10531202 10.1128/iai.67.11.5573-5578.1999 PMC96928

[43] Stahl PD (1992) The mannose receptor and other macrophage lectins. Curr Opin Immunol 4: 49–52. 1317711 10.1016/0952-7915(92)90123-v

[44] Tiemessen MM, Jagger AL, Evans HG, van Herwijnen MJ, John S, et al (2007) CD4+CD25+Foxp3+ regulatory T cells induce alternative activation of human monocytes/macrophages. Proc Natl Acad Sci U S A 104: 19446–19451. 18042719 10.1073/pnas.0706832104 PMC2148309

[45] Gazi U, Martinez-Pomares L (2009) Influence of the mannose receptor in host immune responses. Immunobiology 214: 554–561. 19162368 10.1016/j.imbio.2008.11.004

[46] Mantovani A, Sica A, Sozzani S, Allavena P, Vecchi A, et al (2004) The chemokine system in diverse forms of macrophage activation and polarization. Trends Immunol 25: 677–686. 15530839 10.1016/j.it.2004.09.015

[47] Peach RJ, Bajorath J, Naemura J, Leytze G, Greene J, et al (1995) Both extracellular immunoglobin-like domains of CD80 contain residues critical for binding T cell surface receptors CTLA-4 and CD28. J Biol Chem 270: 21181–21187. 7545666 10.1074/jbc.270.36.21181

[48] Stamper CC, Zhang Y, Tobin JF, Erbe DV, Ikemizu S, et al (2001) Crystal structure of the B7–1/CTLA-4 complex that inhibits human immune responses. Nature 410: 608–611. 11279502 10.1038/35069118

[49] Han TH, Jin P, Ren J, Slezak S, Marincola FM, et al (2009) Evaluation of 3 clinical dendritic cell maturation protocols containing lipopolysaccharide and interferon-gamma. J Immunother 32: 399–407. 19342965 10.1097/CJI.0b013e31819e1773 PMC2832587

[50] Askew D, Chu RS, Krieg AM, Harding CV (2000) CpG DNA induces maturation of dendritic cells with distinct effects on nascent and recycling MHC-II antigen-processing mechanisms. J Immunol 165: 6889–6895. 11120813 10.4049/jimmunol.165.12.6889

[51] Rutkowski R, Moniuszko T, Stasiak-Barmuta A, Kosztyla-Hojna B, Alifier M, et al (2003) CD80 and CD86 expression on LPS-stimulated monocytes and the effect of CD80 and CD86 blockade on IL-4 and IFN-gamma production in nonatopic bronchial asthma. Arch Immunol Ther Exp (Warsz) 51: 421–428. 14692664

[52] Dinarello CA (1994) The interleukin-1 family: 10 years of discovery. Faseb J 8: 1314–1325. 8001745

[53] Botling J, Oberg F, Nilsson K (1995) CD49f (alpha 6 integrin) and CD66a (BGP) are specifically induced by retinoids during human monocytic differentiation. Leukemia 9: 2034–2041. 8609714

[54] Gray-Owen SD, Blumberg RS (2006) CEACAM1: contact-dependent control of immunity. Nat Rev Immunol 6: 433–446. 16724098 10.1038/nri1864

[55] Nagaishi T, Pao L, Lin SH, Iijima H, Kaser A, et al (2006) SHP1 phosphatase-dependent T cell inhibition by CEACAM1 adhesion molecule isoforms. Immunity 25: 769–781. 17081782 10.1016/j.immuni.2006.08.026

[56] Muller MM, Klaile E, Vorontsova O, Singer BB, Obrink B (2009) Homophilic adhesion and CEACAM1-S regulate dimerization of CEACAM1-L and recruitment of SHP-2 and c-Src. J Cell Biol 187: 569–581. 19948503 10.1083/jcb.200904150 PMC2779222

