# Supplementary figures and images for: Moraxella catarrhalis Adhesin UspA1-derived Recombinant Fragment rD-7 Induces Monocyte Differentiation to CD14+CD206+ Phenotype
Source: PLoS One. 2014 Mar 5;9(3):e90999. doi: 10.1371/journal.pone.0090999 (PMC3944954; doi:10.1371/journal.pone.0090999)

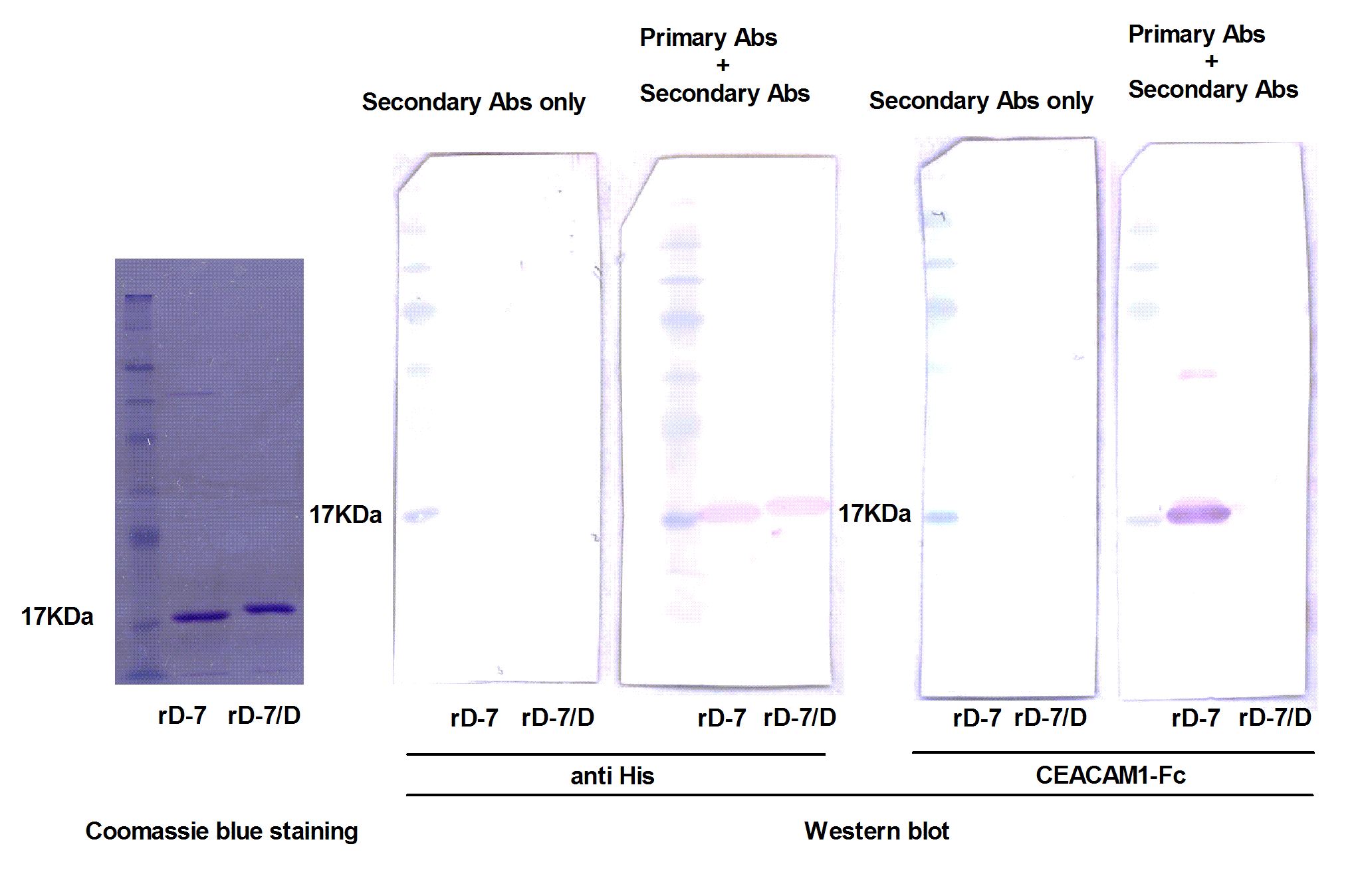

Supplement: Figure S1 — Purity of rD-7 and rD-7/D and their binding to soluble CEACAM1 construct. Purified recombinant molecules were analysed by SDS-PAGE and western blotting. The bands of the expected size (17 kDa) were observed in Coomassie Blue stained gels (left) and in Western blots detected with anti-His tag antibody (middle) and CEACAM1-Fc construct described previously [39]. The 17 kDa band in the rD-7 and rD-7/D were detected with anti-His tag antibody followed by anti-mouse IgG secondary antibody conjugated to alkaline phosphatase. CEACAM1-Fc bound to rD-7 but not rD-7/D. Receptor binding was detected using an anti-human-Fc antibody conjugated to alkaline phosphatase. In the case of both anti-His tag and receptor overlay, alkaline phosphatase conjugates binding was detected by the addition of nitroblue tetrazolium and 5-bromo-4-chloro-3-indoyl phosphate as substrates. (TIF) [file pone.0090999.s001.tif]

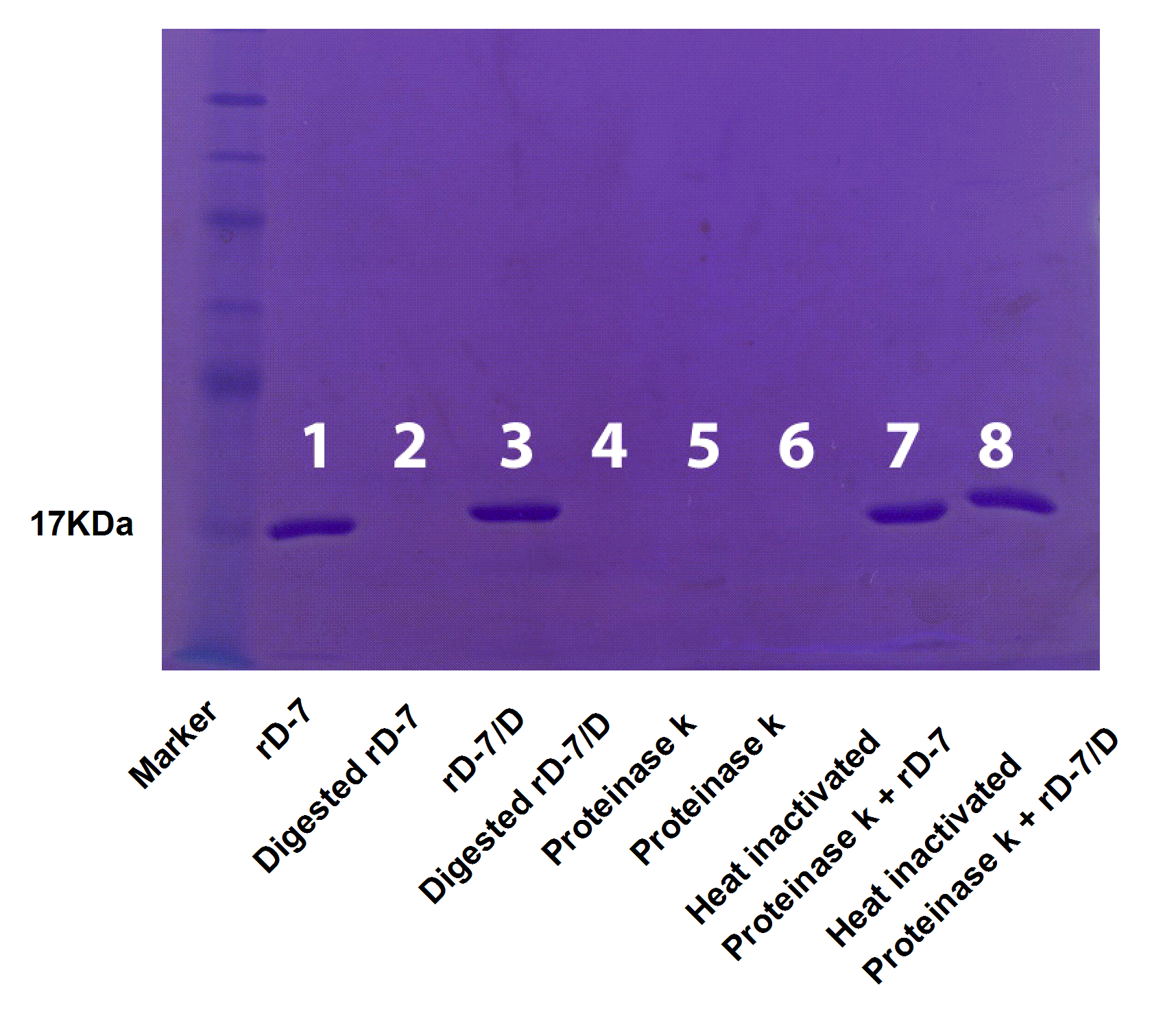

Supplement: Figure S2 — Proteinase K treatment of rD-7 and rD-7/D. Purified samples of rD-7, rD-7/D, or LPS were digested by 200 µg/ml Proteinase K at 37°C for 3 h. The digested samples were heat-inactivated at 95°C for 1 h to inactive Proteinase K and analysed by SDS-PAGE. Sample 1, rD-7; Sample 2, Digested rD-7; Sample 3, rD-7/D; Sample 4, Digested rD-7/D; Samples 5 and 6, Proteinase K; Sample 7, rD-7 with heat-inactivated Proteinase K; Sample 8, rD-7/D with heat-inactivated Proteinase K. (TIF) [file pone.0090999.s002.tif]

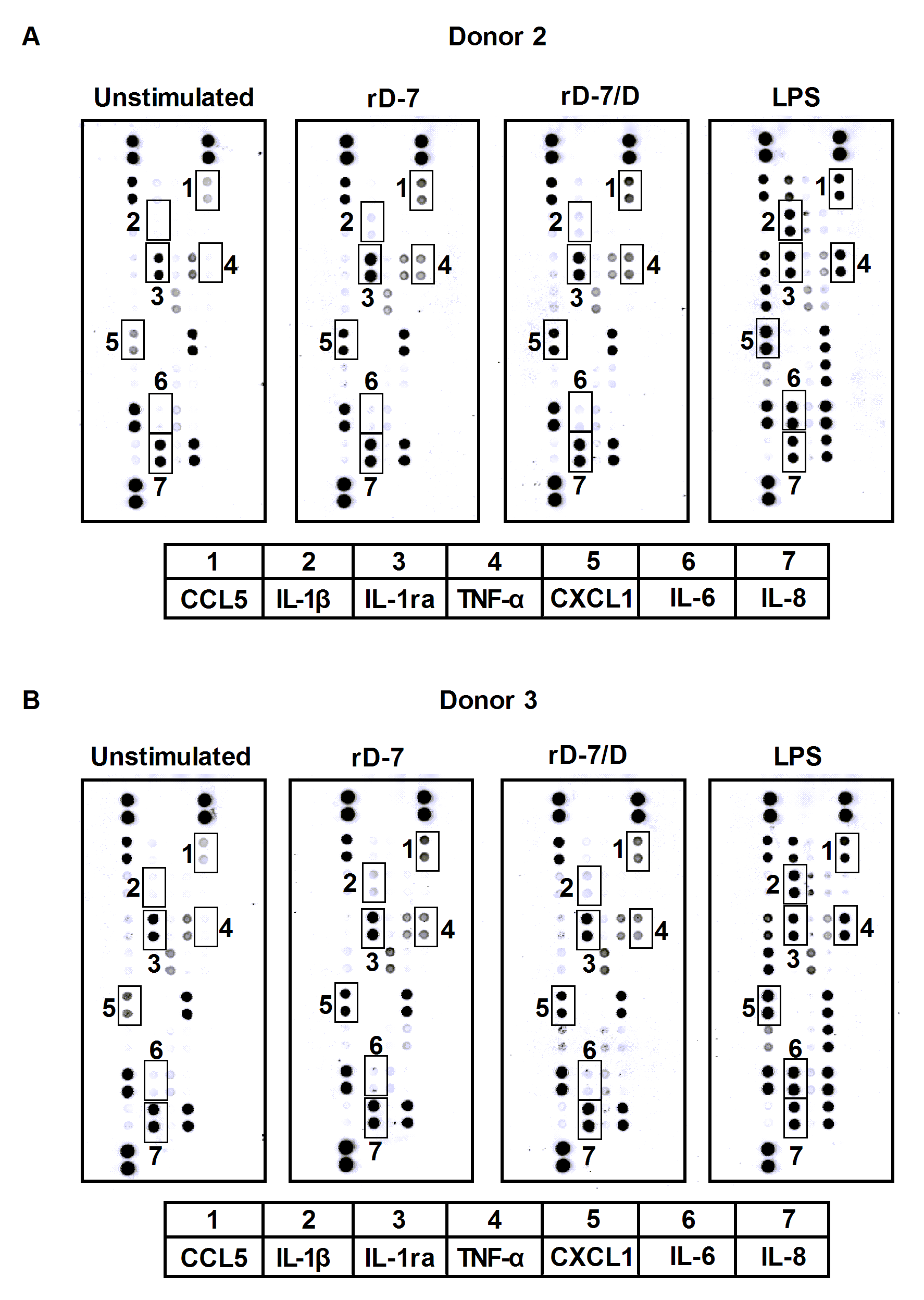

Supplement: Figure S3 — Cytokine and chemokine expression profiles from monocytes modulated by rD-7. Supernatants of monocytes stimulated with rD-7 or rD-7/D from donor 2(A) and donor 3 (B) were used to assess their cytokine and chemokine expression using Proteome Profiler R&D Systems according to the manufacturer’s instructions (data supplementary to Fig. 7). (TIF) [file pone.0090999.s003.tif]
